# Single-stage quadriceps tendon reconstruction with gastrocnemius fascia and knee soft tissue defect reconstruction with medial gastrocnemius flap—A case report

**DOI:** 10.1016/j.ijscr.2020.06.048

**Published:** 2020-06-12

**Authors:** Kresimir Martic, Mladen Vukic, Stipo Matic

**Affiliations:** a Department of Plastic, Reconstructive and Aesthetic Surgery, University Hospital “Dubrava”, Avenija Gojka Suska 6, Zagreb, Croatia; b University of Zagreb, School of Medicine, Department of Surgery, Salata 3, Zagreb, Croatia

**Keywords:** Gastrocnemius fascia, Quadriceps tendon, Reconstruction, Soft tissue defect, Gastrocnemius flap, Case report

## Abstract

**Introduction:**

Quadriceps tendon rupture, although uncommon, is associated with knee joint instability and requires surgical repair. A variety of methods including allografts, synthetic grafts and autologous tendons are used for tendon reconstruction. This work is reported in line with the SCARE criteria (Agha et al., 2018) for case report publication.

**Presentation of case:**

A new method of simultaneous quadriceps tendon reconstruction and soft tissue knee reconstruction in a 38-year-old male patient following trauma to his knee and subsequent multiple surgeries and wound infections.

In a single-stage procedure, gastrocnemius muscle fascia was used for quadriceps tendon reconstruction and medial head of the gastrocnemius muscle flap with split-thickness skin graft was used to cover the soft tissue defect on the anterior aspect of the knee following previous post-operative soft tissue infections and subsequent skin necrosis.

**Discussion:**

The only way to maintain the vitality of the patella was to reconstruct the defect with well-vascularized tissue so we decided to use the medial gastrocnemius muscle flap. To obtain good quality tissue for the quadriceps tendon reconstruction, we decided to use the gastrocnemius muscle fascia to minimise the morbidity of the donor region and to reduce the duration of surgery.

**Conclusion:**

Postoperatively, the patient had an excellent cosmetic outcome with full active extension and limited flexion of the knee joint caused by the preoperative condition. To our knowledge, quadriceps tendon reconstruction using the gastrocnemius muscle fascia has never been reported in English literature and is as valuable as already known reconstruction methods using other tendons or fascia.

## Highlights

• A new method of simultaneous quadriceps tendon reconstruction and soft tissue knee reconstruction.
• Medial head of the gastrocnemius muscle used for reconstruction of the soft tissue knee defect.
• Gastrocnemius muscle fascia used for quadriceps tendon reconstruction.

## 1 Introduction

Simultaneous reconstruction of the extensor mechanism, soft tissue defects and infections management in complex knee injuries presents a great challenge in plastic, reconstructive and aesthetic surgery. This work is reported in line with the SCARE criteria [1] for case report publication.

Local and regional muscle flaps including free flaps are well known to be used to cover soft tissue defects [2]. A variety of methods including allografts, synthetic grafts and autologous tendons, as well as various types of anchors, are used for tendon and tendon insertion reconstructions [[3], [4], [5], [6], [7]]. We report a case of a 38-year-old male patient with a proximal patella and quadriceps tendon insertion loss in conjunction with soft tissue defect above it. The defect was a result of a complicated 8-year-old patella fracture and a patella fixation surgery which resulted in osteomyelitis and proximal patella and soft tissue necrosis.

In a single-stage procedure, the quadriceps tendon was reconstructed using a gastrocnemius muscle fascia and medial head of the gastrocnemius muscle flap with split-thickness skin graft was used to cover the soft tissue defect on the anterior aspect of the knee.

To our knowledge, this type of reconstructive procedure has never been reported in English literature.

## 2 Case report

**Fig. 1:**
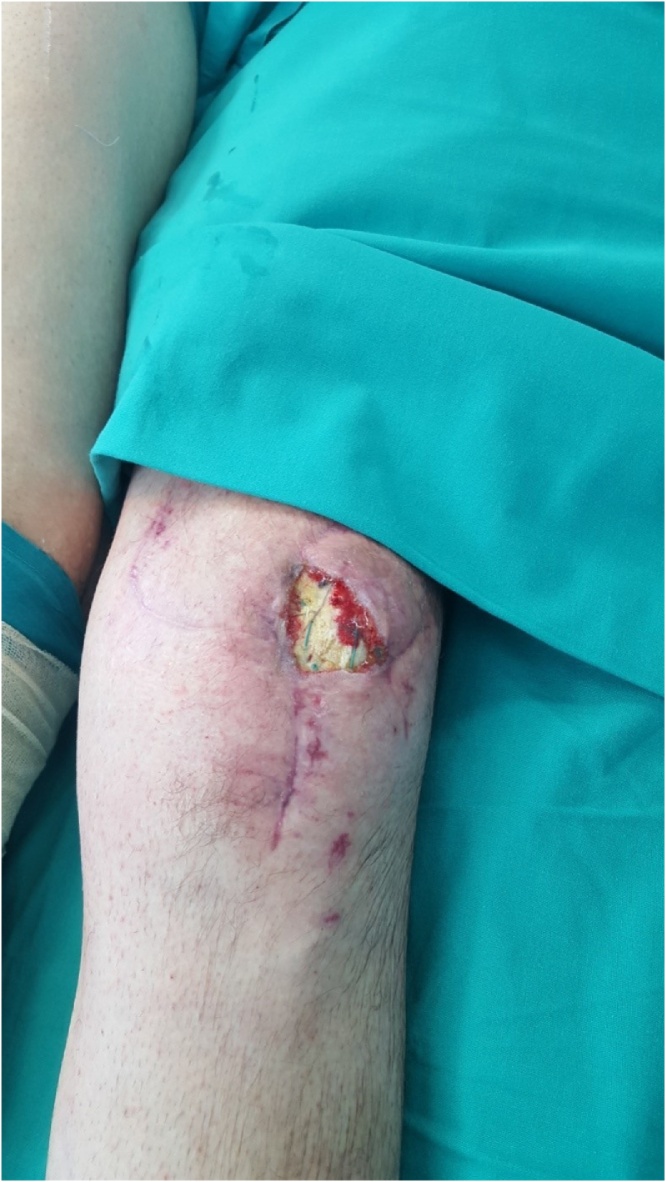
Exposed patella with 4 × 4 cm skin defect and visible patella fixation sutures.

A 38-year-old male patient attended our Plastic Surgery outpatient clinic because of left knee soft tissue defect associated with the exposed proximal patella (Fig. 1). Physical examination of the left knee region revealed exposed left patella with questionable vitality and 4 × 4 cm skin defect with visible patella fixation sutures.

Eight years ago, the patient was involved in a traffic accident driving a motorcycle and sustained left patella fracture. He underwent surgery which was not carried out in our hospital. Few months following the initial surgery, osteosynthesis metalwork was removed because of the infection. After the metalwork removal, the wound healing was complicated with wound dehiscence. Therefore, negative pressure wound therapy, antibiotics and physical therapy were all used for wound healing.

The wound healed with elongated callus formation at the fracture site and the patient was able to straight leg raise. Two months before the patient presented in our clinic, his orthopaedic surgeon performed the resection of the callus and patella osteosynthesis. The procedure was complicated by the proximal patella necrosis and soft tissue necrosis. After proximal patella debridement, resection and reinsertion of the quadriceps muscle tendon with transoseal sutures was performed and the exposed bone was covered with a local flap. Post-operatively, the local flap used to cover the soft tissue defect became necrotic, so it was debrided and negative pressure wound therapy was applied.

To cover the soft tissue defect and to preserve the remaining patella, we planned to use medial gastrocnemius flap with medial sural artery and vein pedicle. Instead of well-known reconstructions of quadriceps muscle tendon using fascia lata [8] or semitendinosus tendon [9], we decided to use gastrocnemius muscle fascia.

**Fig. 2:**
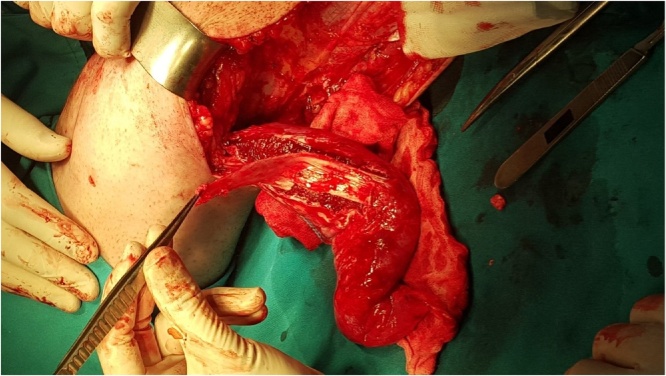
Harvesting gastrocnemius muscle fascia.

**Fig. 3:**
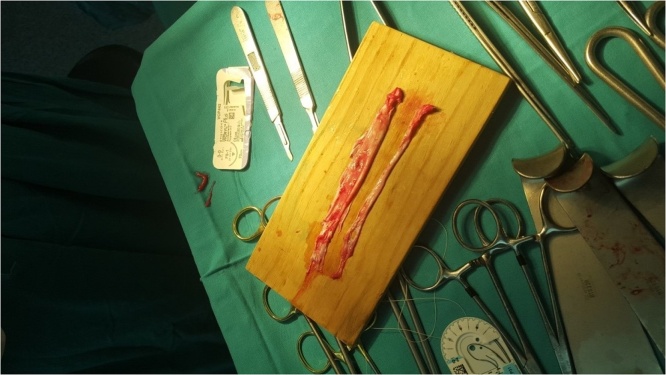
Gastrocnemius muscle flap fascia.

**Fig. 4:**
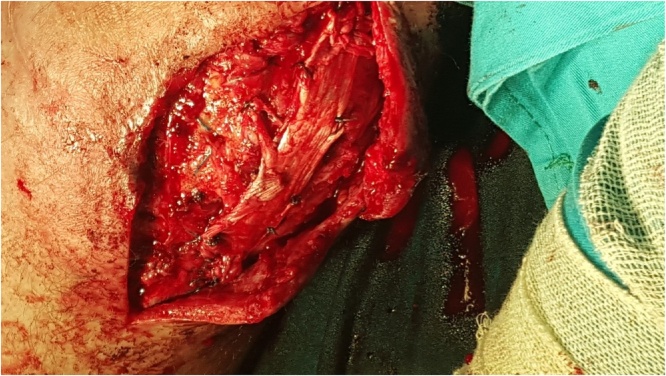
Patella tendon reinforced with the remaining fascia.

After skin-sparing debridement of the anterior aspect of the left knee, medial head of gastrocnemius muscle was fully mobilized by cutting free gastrocnemius muscle origin and insertion. The gastrocnemius muscle fascia was harvested and a tubular structure was made out of it (Fig. 2, Fig. 3). A transverse tunnel (2.5 mm in diameter) was made through the patella. The tubular fascia was pulled through the formed tunnel and, more proximally, fixed to the remaining part of the quadriceps femoris tendon. Furthermore, the patellar tendon was reinforced on the anterior aspect with the remaining gastrocnemius muscle fascia (Fig. 4).

The soft tissue defect was reconstructed with a gastrocnemius muscle flap and the flap was covered with a split-thickness skin graft (Thiersch). Redon drain was put in the donor site and negative pressure wound therapy dressing was applied to the knee wound.

Postoperatively, the patient had no complications. Negative pressure wound therapy dressing was removed on the third post-op day with skin graft acceptance and vital muscle flap. On the 10th post-op day wound looked healthy and above knee backslab was applied. One month following the surgery, the patient started having passive physiotherapy and three months after the surgery active physiotherapy was started with gradually putting weight on his left leg.

**Fig. 5:**
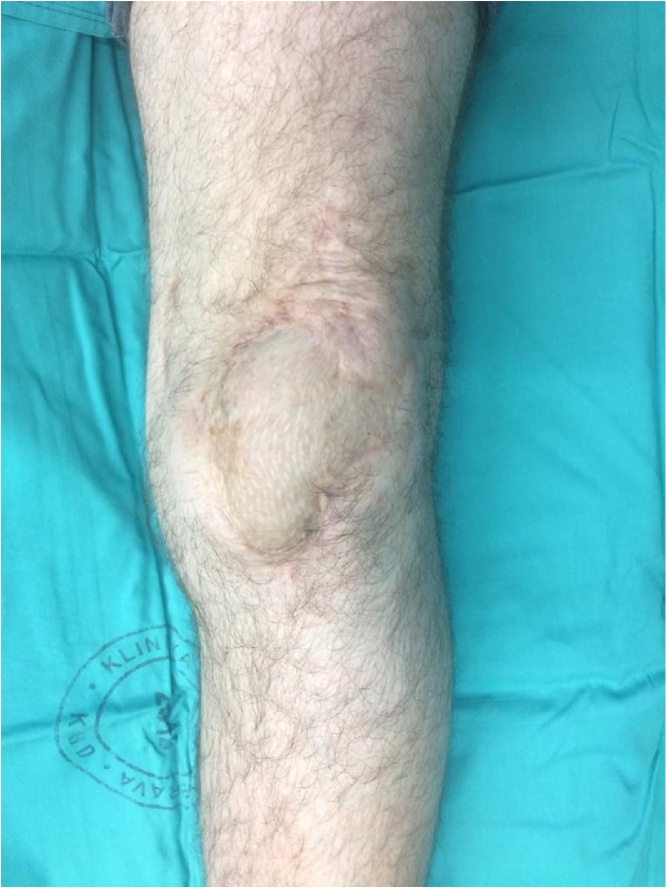
Twelve months after the surgery.

The patient was last seen in our clinic 12 months after the surgery and he had full active knee extension and limited flexion up to 110° with excellent cosmetic outcome (Fig. 5).

## 3 Discussion

Our patient required thorough approach because of a complex skin and soft tissue defect with exposed patella associated with chronic osteomyelitis. Furthermore, the quadriceps femoris tendon reconstruction made the reconstruction procedure even more difficult and challenging.

The only way to maintain the vitality of the patella was to reconstruct the defect with well-vascularized tissue so we decided to use the medial gastrocnemius muscle flap [6,7,10]. The purpose of using medial gastrocnemius muscle flap was not just to cover the exposed bone but also to solve the problem of postoperative necrosis and infection in the patella region. Furthermore, we had a dilemma whether to do the quadriceps tendon reconstruction simultaneously with the knee soft tissue reconstruction or to do it at a later stage once the wound has healed having in mind that single-stage reconstruction has good functional results and that, on the other hand, multiple-stage reconstruction is time-consuming and produces considerable suffering for patients [10].

With the view to avoid further surgery and considering the patient’s age and usual activity, we decided to perform both procedures in one sitting. The decision was made to perform the reconstruction using a patient’s tissue rather than using foreign material to achieve the best possible outcome, especially when taking into consideration the local findings and complexity of the case.

To obtain good quality tissue for the quadriceps tendon reconstruction, we decided to use the gastrocnemius muscle fascia, to minimise the morbidity of the donor region and also to reduce the duration of the surgery. We find this approach to quadriceps tendon reconstruction to be logical and natural in complicated cases such as this one where it is necessary to use a muscle flap and at the same time minimise additional trauma to the soft tissue. The required length and strength of the tissue for the quadriceps tendon reconstruction was obtained without further damage to the surrounding tissue.

This modified method of knee soft tissue defect and quadriceps tendon reconstruction has not been described in the literature so far. A similar method was described by Kim R.H. et al., who reconstructed patella tendon using Achilles tendon which they passed through a tunnel in the patella and used medial gastrocnemius muscle flap to cover the soft tissue defect above the patella [11].

Rhomberg et al. described the reconstruction of quadriceps femoris and patella tendons with a deep aponeurotic gastrocnemius muscle fascia in partial tendon defects and in the full tendon defects they also used the superficial layer of the Achilles tendon [3,12]. The described reconstruction methods are less conservative and use a part of Achilles tendon, which in our case was not used at all.

Our patient was last seen in clinic 12 months after the surgery and he had a full active extension of the knee joint. The flexion was limited up to 110° which is more than acceptable considering the preoperative findings. Furthermore, the patient was able to walk normally, climb up the stairs but had difficulties running and walking down the stairs. On the other hand, the cosmetic outcome was excellent and the patient was more than happy with the results.

## 4 Conclusion

The outcome of our approach confirms our expectations that this new method of quadriceps tendon reconstruction is as valuable as already known reconstruction methods using other tendons or fascia. This method has the potential of becoming the standard in complicated cases which include both tendon and soft tissue defect reconstruction in the knee area.

## Declaration of Competing Interest

All authors declare no conflict of interest related to this case report.

## Funding

We have nothing to declare.

## Ethical approval

Written informed consent was obtained from the patient for publication of this case report and accompanying images. A copy of the written consent is available for review by the Editor-in-Chief of this journal on request.

## Consent

Written informed consent was obtained from the patient for publication of this case report and accompanying images. A copy of the written consent is available for review by the Editor-in-Chief of this journal on request.

## Author contribution

Kresimir Martic (main surgeon, case report concept and design).

Mladen Vukic (writing the paper-original draft).

Stipo Matic (writing the paper-review and editing.

## Registration of research studies

1. Name of the registry: N/A.
2. Unique identifying number or registration ID: N/A.
3. Hyperlink to your specific registration (must be publicly accessible and will be checked): N/A.

## Guarantor

Kresimir Martic, PHD MD.

Mladen Vukic, MD.

Stipo Matic, MD.

## Provenance and peer review

Not commissioned, externally peer-reviewed.
